# Endoscopic removal of a coin embedded in the esophagus of a 2-year-old male

**DOI:** 10.1016/j.vgie.2026.03.008

**Published:** 2026-04-02

**Authors:** Alex I. Halpern, Mikael Petrosyan, Timothy D. Kane

**Affiliations:** Department of General and Thoracic Surgery, Children's National Hospital, Washington, District of Columbia, USA

## Abstract

**Background and Aims:**

Endoscopic esophageal foreign body removal is a common procedure performed by pediatric gastroenterologists and surgeons. However, when foreign bodies are embedded in the esophageal wall, traditional treatment has instead involved surgical resection. As recently described, advanced endoscopic techniques enable endoscopic retrieval of embedded esophageal foreign bodies.

**Methods:**

We present a case of endoscopic retrieval of a coin embedded in the esophagus of a 2-year-old male. The patient presented with wheezing and radiograph findings consistent with an esophageal foreign body. Endoscopy was performed, but the procedure was aborted after the coin was identified to be completely embedded within the mucosa of the esophageal wall. A CT scan then confirmed the embedded coin.

**Results:**

A decision was made to perform a repeat endoscopy, with conversion to thoracotomy if the coin could not be successfully removed endoscopically. On repeat endoscopy, the coin was visualized, although its superior edge was surrounded by heaped-up mucosa. Mucosotomy and myotomy were necessary to fully expose the coin, which was then removed. The patient's diet was advanced postoperatively, and he was discharged after an esophagram showed no evidence of a leak.

**Conclusions:**

Providers should consider endoscopic removal of embedded esophageal foreign bodies for pediatric patients.

## Introduction

Endoscopic esophageal foreign body removal is a common procedure performed by pediatric gastroenterologists and surgeons.^1^^,^^2^ However, when foreign bodies are embedded in the esophageal wall, traditional treatment has instead involved surgical resection, which is associated with significant morbidity.^3^^,^^4^ As recently described, advanced endoscopic techniques enable endoscopic retrieval of embedded esophageal foreign bodies.^5^, ^6^, ^7^, ^8^, ^9^ We present a case of endoscopic retrieval of a coin embedded in the esophagus of a 2-year-old male (Video 1, available online at www.videogie.org).

## Case presentation

**Figure 1:**
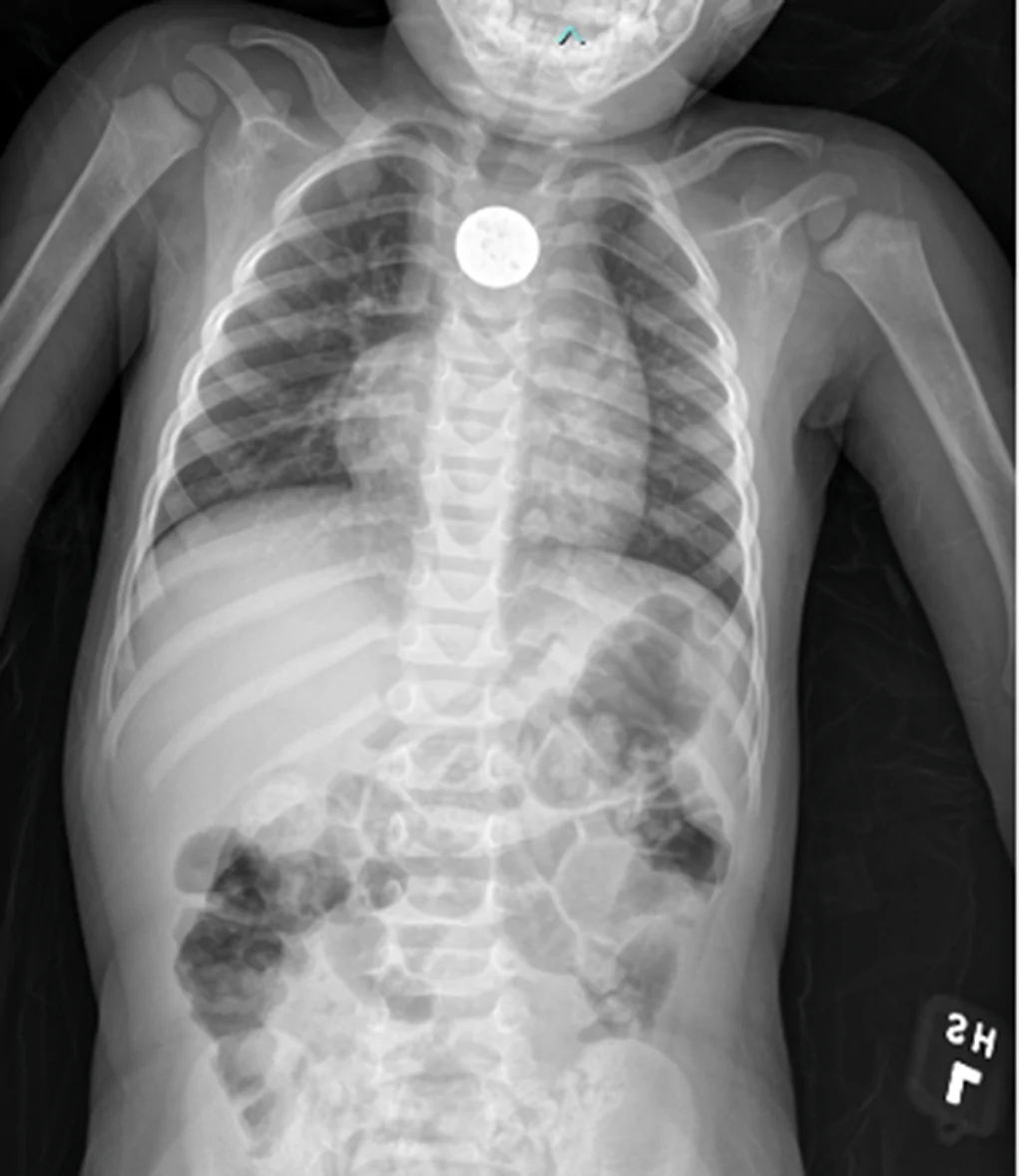
Preoperative radiograph demonstrating an airway foreign body.

A 2-year-old-male presented to the emergency department with 1 day of wheezing. The patient was hemodynamically stable and able to handle his own secretions but was uncomfortable. The patient had an unknown timeframe of ingestion history and radiographic findings consistent with an esophageal foreign body (Fig. 1).

**Figure 2:**
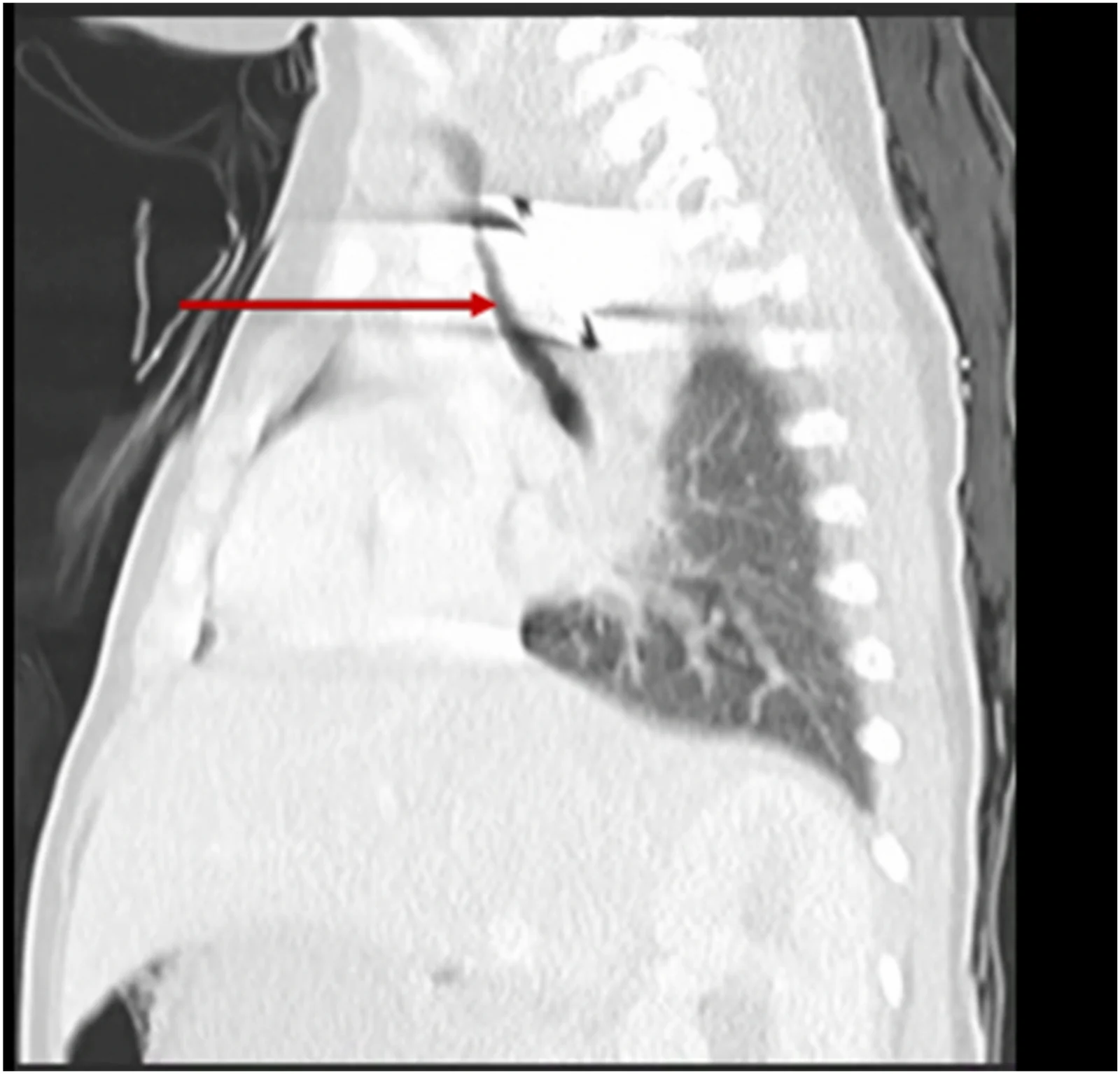
Preoperative CT scan demonstrating a metallic foreign body (indicated by the *red arrow*) within the esophageal wall.

Flexible endoscopy was performed, but the procedure was aborted after a coin was identified to be completely embedded beneath the mucosa in the proximal esophageal wall. EUS was not available during this case. A CT scan then confirmed the embedded coin (Fig. 2). After discussion with the patient's guardian, a decision was made to perform a repeat endoscopy, with conversion to thoracotomy if the coin could not be successfully removed endoscopically.

**Figure 3:**
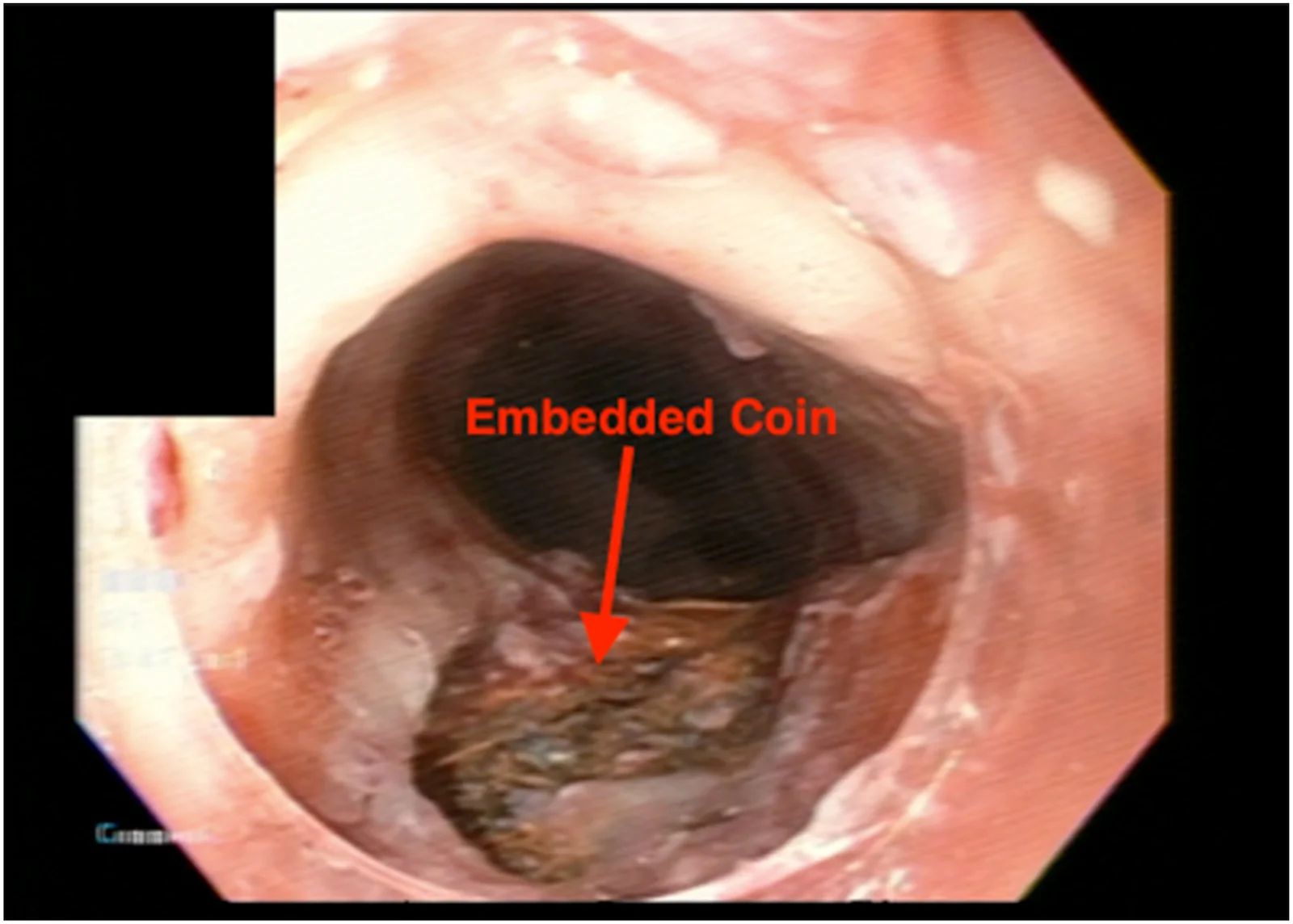
Coin visualized on endoscopy surrounded by heaped-up mucosa.

**Figure 4:**
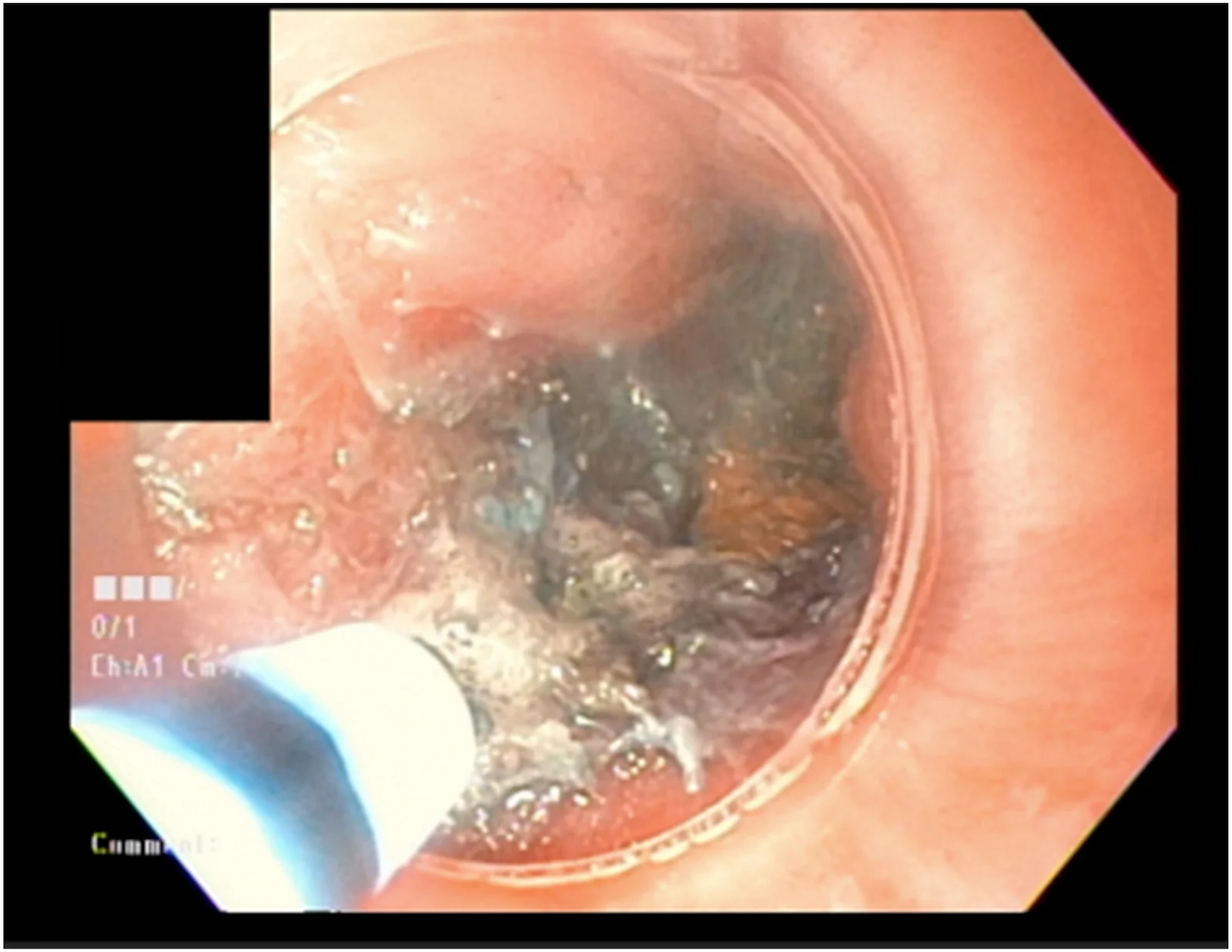
Mucosotomy and myotomy performed with an electrosurgical knife (HybridKnife; Erbe, Tübingen, Germany) to expose the coin.

**Figure 5:**
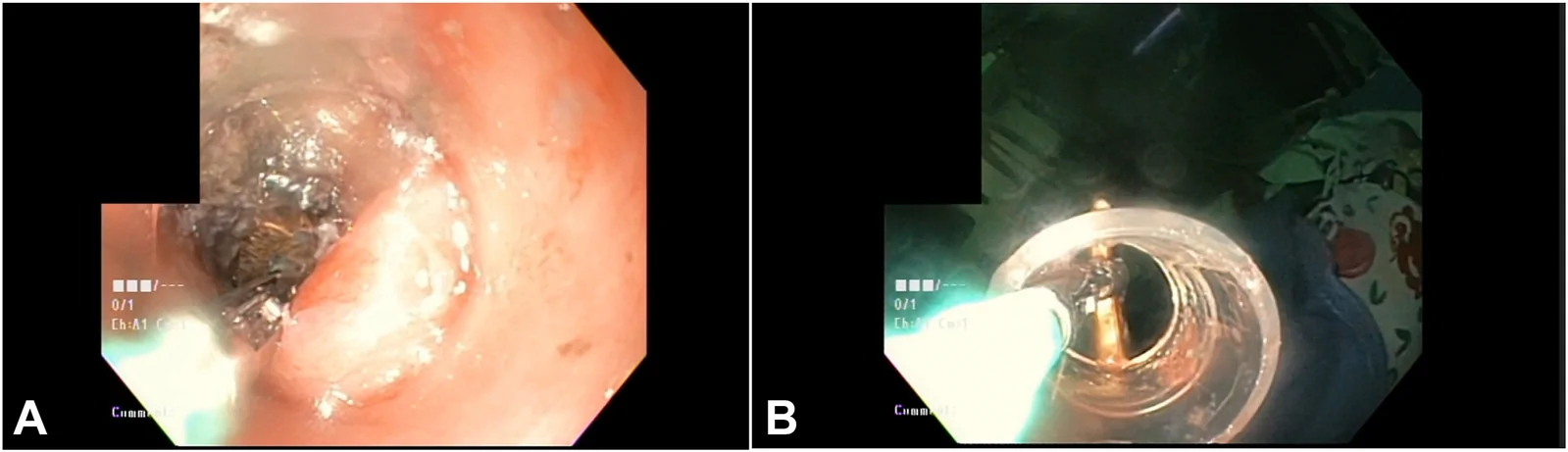
The coin was grasped with a rat-tooth grasper **(A)** and removed **(B)**.

**Figure 6:**
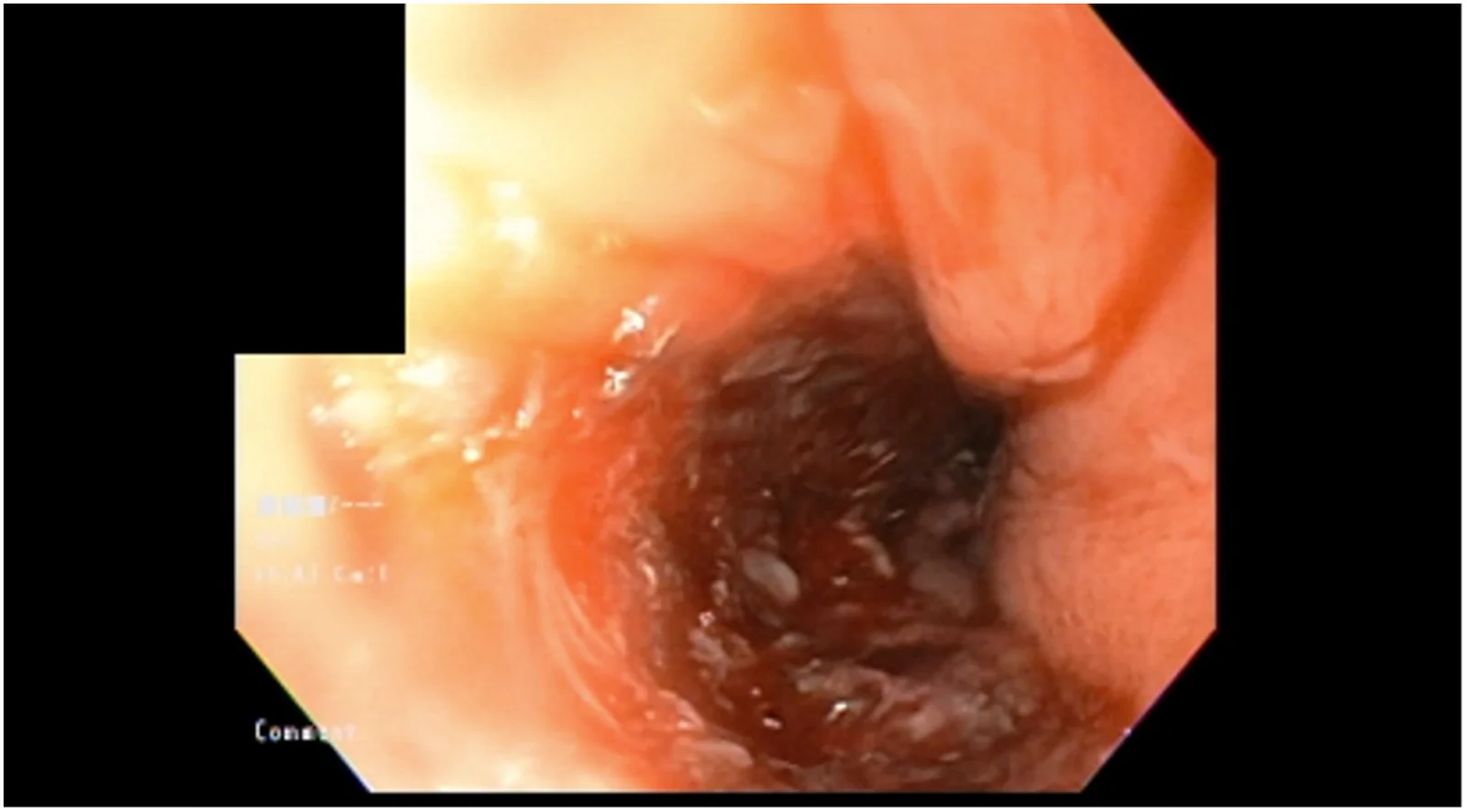
Location of the foreign body after removal, demonstrating significant edema without full-thickness injury.

On endoscopy, the coin was visualized embedded in the esophageal wall, with its superior edge surrounded by heaped-up mucosa (Fig. 3). After initial attempts to remove the coin were unsuccessful, 1.5-cm mucosotomy and myotomy were performed using an electrosurgical knife (HybridKnife; Erbe, Tübingen, Germany) to fully expose the coin (Fig. 4). The coin was then grasped with forceps and removed (Fig. 5). There was significant edema at the location of the foreign body, without evidence of full-thickness injury (Fig. 6). The mucosa was left open to heal through secondary intention. The endoscope was not insinuated past the mucosal defect so as not to further traumatize the area and risk full-thickness injury. Notably, a submucosal injection was not used before the mucosotomy and myotomy, which could have potentially further increased the safety of dissection around the embedded coin. In addition, EUS and submucosal tunnel were not performed because we were able to visualize part of the coin, and a submucosal tunnel was not needed to reach it.

**Figure 7:**
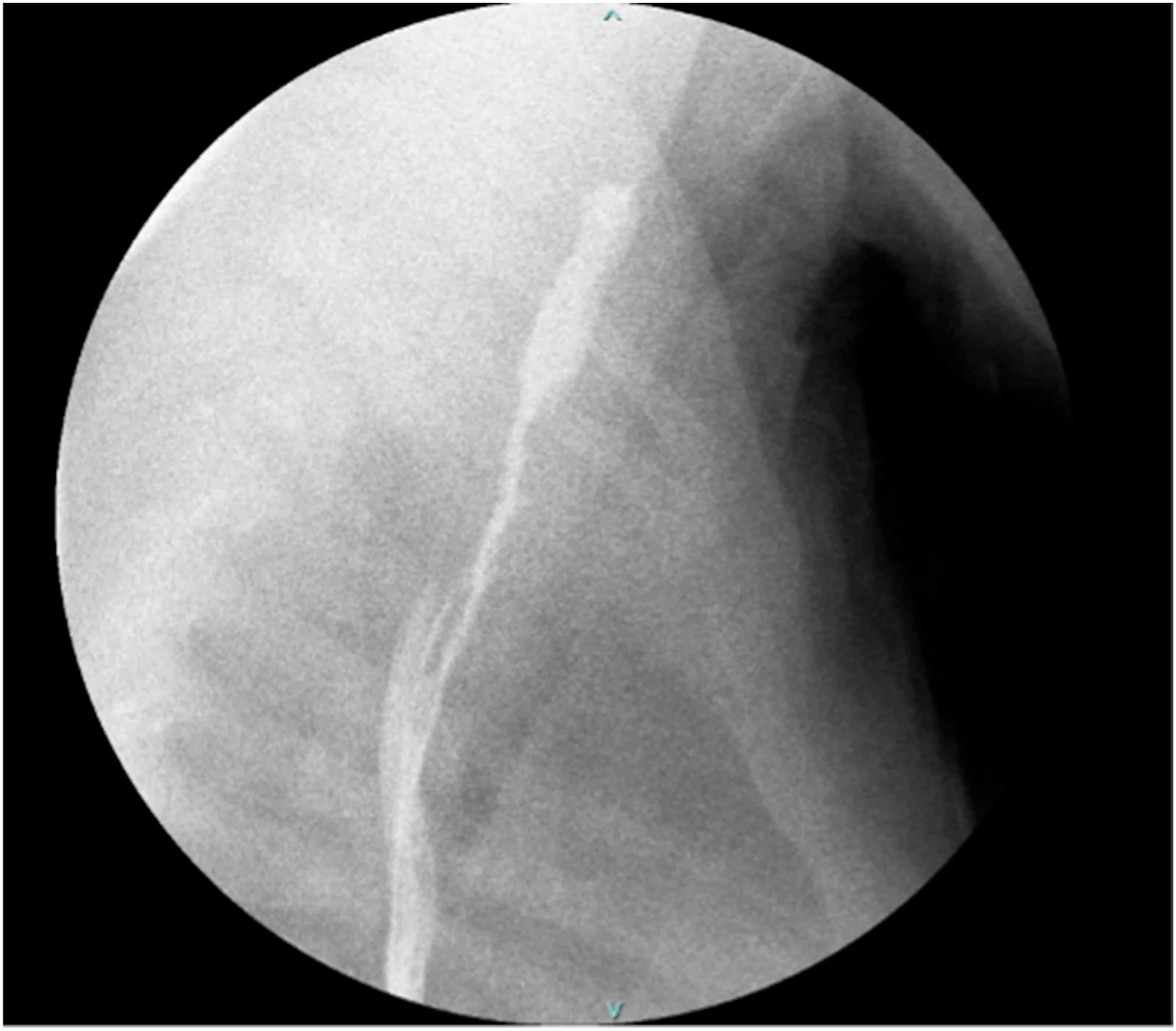
Postoperative esophagram demonstrating expected esophageal narrowing and no evidence of a leak.

After surgery, the patient was started on pantoprazole and ampicillin/sulbactam. He was initially kept nil per os, with diet advancement to full liquids on postoperative day 1. The patient was discharged on postoperative day 2 after an esophagram showed expected narrowing without evidence of a leak (Fig. 7). At discharge, the patient was prescribed pantoprazole for 30 days. Although he was lost to follow-up, he had not presented to the hospital with symptoms in over 9 months.

## Conclusion

Embedded esophageal foreign bodies pose a challenge for clinicians and have historically been removed via open surgery with great morbidity. With recent advances in endoscopy, purely endoscopic removal of embedded esophageal foreign bodies for pediatric patients is feasible and safe.

## Patient consent

All attempts have been exhausted in trying to contact the parents or guardian for the purpose of attaining their consent to publish this case. No identifying patient information is included.

## Disclosure

All authors disclosed no financial relationships.

## Supplementary data

Video 1 Case presentation.
